# Functional Lipids in Autoimmune Inflammatory Diseases

**DOI:** 10.3390/ijms21093074

**Published:** 2020-04-27

**Authors:** Michele Dei Cas, Gabriella Roda, Feng Li, Francesco Secundo

**Affiliations:** 1 Department of Health Sciences, Università degli Studi di Milano, 20142 Milan, Italy; 2 Department of Pharmaceutical Sciences, Università degli Studi di Milano, 20133 Milan, Italy; 3 State Key Laboratory of Virology, Wuhan Institute of Virology, Chinese Academy of Sciences, Wuhan 430071, China; 4 Istituto di Scienze e Tecnologie Chimiche “Giulio Natta”, Consiglio Nazionale delle Ricerche, 20131 Milan, Italy

**Keywords:** inflammation, lipids, lysoglycerophospholipids, sphingolipids, endocannabinoids

## Abstract

Lipids are apolar small molecules known not only as components of cell membranes but also, in recent literature, as modulators of different biological functions. Herein, we focused on the bioactive lipids that can influence the immune responses and inflammatory processes regulating vascular hyperreactivity, pain, leukocyte trafficking, and clearance. In the case of excessive pro-inflammatory lipid activity, these lipids also contribute to the transition from acute to chronic inflammation. Based on their biochemical function, these lipids can be divided into different families, including eicosanoids, specialized pro-resolving mediators, lysoglycerophospholipids, sphingolipids, and endocannabinoids. These bioactive lipids are involved in all phases of the inflammatory process and the pathophysiology of different chronic autoimmune diseases such as rheumatoid arthritis, multiple sclerosis, type-1 diabetes, and systemic lupus erythematosus.

## 1. Introduction: Lipids and Inflammation

Inflammation is an immune response that occurs following infections, cellular insults, or tissue insults and that can be spontaneously exhausted after the elimination of the damage. It includes an extensive network of cellular and molecular processes, in which a multitude of preformed or newly synthesized mediators are arranged to obtain specific responses. However, protracted and uncontrolled immune responses can lead to chronic inflammation, irreparable tissue damages, and chronic diseases. Uncontrolled immune responses also occur in many common autoimmune diseases, including type 1 diabetes, multiple sclerosis (MS), psoriasis, inflammatory bowel disease (IBD), and Grave’s disease.

**Figure 1:**
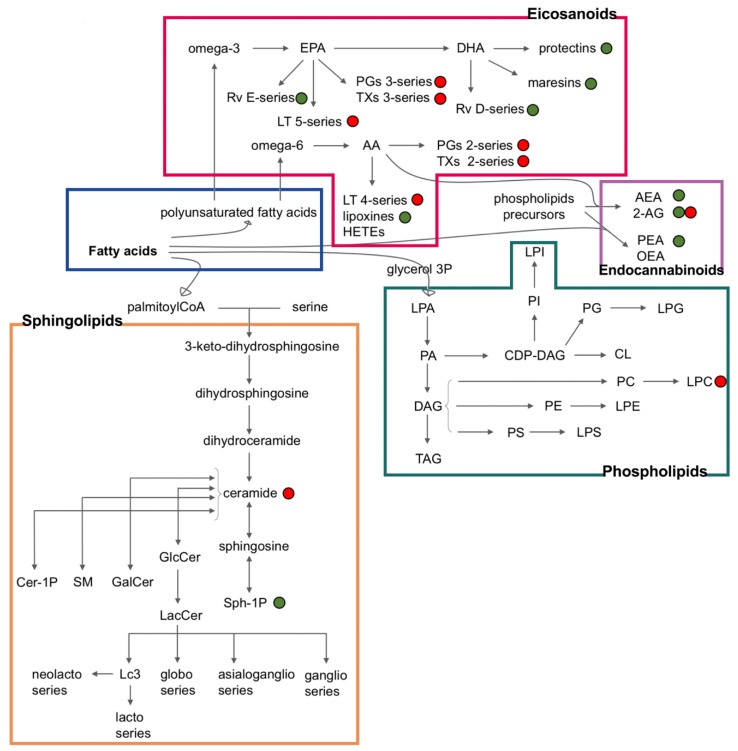
Inflammatory lipids and their interconnections. The green points indicate lipids with anti-inflammatory properties, whereas the red points indicate lipids with pro-inflammatory properties [5,6,7,8,9,10,11,12,13,14,15,16,17,18,19,20,21,22,23,24,25,26,27,28,29,30,31,32,33,34,35,36,37]. FA: fatty acids and their derivatives; PUFA: polyunsaturated fatty acids containing both endocannabinoids and eicosanoids; AA: arachidonic acid; EPA: eicosapentaenoic acid; DHA: docosahexaenoic acid; GL: glycerophospholipids; LTs: leukotrienes; PGs: prostaglandins; TXs: thromboxanes; Rv: resolvins; AEA: anandamide; 2-AG: 2-arachidonoilglycerol; PEA: palmitoylethanolamide; OEA: O-arachidonoylethanolamine; LPA: lysophoshatidic acid; PA: phosphatidic acid; DAG: diacyglicerols; TAG: triacylglicerols; CDP-DAG: cytidine diphosphate diacylglycerol; CL: cardiolipins; PI: phosphatidyl inositols; LPI: lysophosphatidylinositols; PG: phosphatidyl glycerols; LPG: lysophosphatidylglycerols; PE: phosphatidylethanolamines; LPE: lysophosphatidylethanolamines; PC: phosphatidyl cholines; LPC: lysophosphatidylcholines; PS: phosphatidyl serines; LPS: lysophosphatidylserines; GlcCer: glucosylceramide; LacCer: lactosylceramide; S1P: sphingosine 1-phosphate; GalCer: galactosylceramide; SM: sphingomyelins; Cer-1P: ceramide 1-phosphate.

Endogenous bioactive lipids play a pivotal role in inflammatory processes and in triggering, coordinating, and confining immunity by regulating hypervascular reactivity, pain, leukocyte trafficking, and clearance [1,2,3,4]. Moreover, the accrual of inflammatory lipids can contribute to the transition from acute to chronic inflammation (Figure 1) [1].

Bioactive lipids can be divided into different families depending on their structure or biochemical function: eicosanoids, specialized pro-resolving mediators, lysoglycerophospholipids, sphingolipids, and endocannabinoids [3].

## 2. Lipids Involved in Inflammatory Responses

### 2.1. Eicosanoids

This well-known family of bioactive lipids includes a wide range of derivatives of fatty acids with a 20-carbon chain, such as arachidonic acid (AA), eicosapentaenoic acid (EPA), and docosahexaenoic acid (DHA). Eicosanoids are prevalently produced from AA, which can be released from membrane phospholipids primarily by phospholipase A_2_ and secondarily by phospholipase C. Three different enzymes drive the biosynthesis of eicosanoids: (1) cyclooxygenases 1 and 2 (COX-1/2) produce the class of prostanoids that comprises prostaglandins (PGs), prostacyclins, and thromboxanes (TXs); (2) lipoxygenases (5/12/15-LOX) generate leukotrienes (LTs), hydroxy-eicosatetraenoids, and lipoxins; and (3) P450 epoxygenase generates hydroxy-eicosatetraenoic acids (HETEs) and epoxy-eicosatrienoids [38,39]. Eicosanoids are divided into omega-6 and omega-3 families depending on the position of unsaturations of their precursor. The pro-inflammatory omega-6 family is derived from AA, whereas the anti-inflammatory omega-3 family is derived from EPA and DHA.

Eicosanoids are well-recognized for initiating inflammation and for controlling vascular tone, platelet aggregation, pain perception, ovulation, and embryo implantation. In general, the omega-6 eicosanoids are pro-inflammatory, whereas the omega-3 eicosanoids do not promote the inflammatory events and can also be anti-inflammatory and pro-resolving [40,41].

In particular, five mechanisms are related to inflammation induced by PGs: (1) boosting the release of pro-inflammatory cytokines [41,42]; (2) enhancing the innate immunity [43]; (3) activating the T-helper cells, TH1-related TH17 [44]; (4) contributing to leukocyte recruitment [41]; and (5) increasing the expression of pro-inflammatory genes such as NF-κB [45].

The leading roles of LTs in acute inflammation are to induce edema and to maintain an ongoing inflammatory status by acting as chemoattractants for neutrophils, macrophages, eosinophils, and TH_17_ lymphocytes. TXs and prostacyclins have prevalent functions of vasoconstriction and vasodilatation, respectively [1,3,46]. Epoxyeicosatrienoic acids (EETs) are synthesized from AA by cytochrome P450 epoxygenases. They modulate vasorelaxation, anti-inflammation—by the suppression of NF-kB activation—and fibrinolysis. They can be converted into the less active dihydroxyeicosatrienoic acids (DHETs) by soluble epoxide hydrolase [47].

### 2.2. Sphingolipids

Sphingolipids are amino alcohols synthesized de novo from the condensation of serine and acyl-CoA. They are involved in a multitude of pathophysiological functions [48], such as regulation of apoptosis [49,50], proliferation [51], differentiation [52], autophagy [53,54], invasiveness [55,56], modification of signaling cascade [57,58], and mediation of inflammatory responses by cytokines [59]. In particular, ceramide and sphingosine promote apoptosis via different pathways, which involves the catalytic activity of Bcl-2, protein kinase C, protein phosphatases 1–2, and proteases. In contrast with ceramide, which is predominantly pro-apoptotic [60], sphingosine-1-phosphate (S1P) is mainly an anti-apoptotic messenger [61] that can modulate mitogenesis, cell migration, cytoskeletal rearrangement, and angiogenesis [54,62]. The phosphate forms of sphingolipids are notably related to inflammation, where S1P acts on either COX-2 or NF-kB and ceramide-1P acts on phospholipases A2 [63,64]_._

### 2.3. Endocannabinoids

Endocannabinoids are a group of molecules that can bind and activate cannabinoid receptors (CB1 and CB2) in the same way as the tetrahydrocannabinol (THC), the main psychoactive component of *Cannabis sativa*. These lipid mediators share a cannabimimetic action but have different chemical structures, which have chemical functionalities such as amides, esters, or ethers of long-chain polyunsaturated fatty acids. The most studied molecules of this class are anandamide (AEA), 2-arachidonoilglycerol (2-AG), 2-AG-ether, O-arachidonoylethanolamine, arachidonoyldopamine, and palmitoylethanolamide (PEA). These are known as potent immunoregulatory, endocrine, and inflammatory modulators [65,66]. In particular, AEA and PEA have anti-inflammatory properties [67,68], whereas 2-AG has both pro- and anti-inflammatory properties [69,70,71]. Thus, some dysfunctions in tissue homeostasis and chronic inflammatory status were related to changes in the concentrations, metabolism, and receptors of endocannabinoids [72].

### 2.4. Lysoglycerophospholipids

These lipids are asymmetrically distributed in the plasma membrane. In particular, phosphatidylethanolamine, phosphatidylserine, and phosphatidylinositol are mainly located in the inner membrane leaflet, whereas phosphatidylcholine is located in the outer membrane leaflet. Lysoglycerophospholipids contain glycerol backbones linked to two long fatty acid chains and a polar head comprising a phosphate modified with ethanolamine, choline, inositol, or serine [73]. The most active forms of lysoglycerophospholipids derive from the hydrolytic removal of one of the fatty acids from the membrane phospholipids. This hydrolysis process affords lysophosphatidylcholines (LPC), lysophosphatidylinositols (LPI), lysophosphatidylethanolamines (LPE), lysophosphatidylserines (LPS), and lysophosphatidic acid (LPA). Lysophospholipids act as signal molecules in the inflammatory cascade and in the essential processes of cellular and tissue life, such as plasma membrane shaping, cell growth, and cell death [33]. LPC and LPA modulate the immune response by controlling the distribution, trafficking, and activation of immune cells [33,74]. Therefore, they have been linked with different inflammatory diseases such as diabetes, obesity [75], atherosclerosis, cancer [76], and rheumatoid arthritis (RA) [77].

## 3. The Connection between Lipids and Some Related Inflammatory Diseases

Uncontrolled chronic inflammation occurs in autoimmune inflammatory diseases (AIDs) and is induced by the overreaction of the immune system to the organs or tissues of the body. The lipid-mediated inflammation and pathogenesis of AIDs have been investigated, and the results are shown in Table 1 and summarized as follows.

### 3.1. Rheumatoid Arthritis

RA is a systemic AID of the joints that is characterized by excessive synovial and joint inflammation, which contribute to the deterioration of bone and cartilage. Eicosanoids are implicated in the development of synovitis and the disintegration of the joints in inflammatory arthritis. The pathogenesis of rheumatoid and psoriatic arthritis was proposed to arise from an imbalanced regulation of pro- and anti-inflammatory eicosanoids [5,78]. In particular, the anti-inflammatory EPA-derived eicosanoids, including 11-HEPE, 12-HEPE, and 15-HEPE, were up-regulated. This increase was suggested to occur to equalize the inflammation induced by AA-derived eicosanoids [5]. On the contrary, another study demonstrated a decrease in some pro-resolving lipid mediators in the circulating plasma [28]. 15-(S)-HETE and 13-HODE stimulated the expression of placenta growth factor, which plays an essential role in RA. The up-regulation of placenta growth factor was related to COX-2 activity as well as PGE2 levels and membrane-bound receptors expression in eicosanoids (EP1, EP2, EP3, and EP4) [17,79]. In a murine model affected by RA, the levels of the different molecules (e.g., PGE2, PGD2, PGF2α, and TXB2) related to COX and LOX pathways [6,78] were significantly higher than the control. In contrast, the levels of metabolites 5-HpETE and LTD4 were lower than the control [6]. In addition, the early phase of RA was characterized by the synthesis of PGD2 within the joint, with the peak of expression being reached in the later stages. Moreover, serum PGD2 levels increased throughout the arthritic process, thus taking part in the anti-inflammation activity [80]. In RA, oxidative stress is accompanied by inflammation, and these were monitored by the increase in the plasma levels of 8-iso-prostaglandin F2α and 15-keto-dihydro-PGF(2α) [81,82].

The pathophysiology of RA comprises synovial inflammation, hyperplasia, and cartilage degradation; moreover, it could be linked to the endocannabinoid system. The activation of CB2 can alleviate the disease by inhibiting not only the development of autoantibodies, pro-inflammatory cytokines, and matrix metalloproteinases but also bone degradation, T-cell-mediated immune response, and fibroblast-like proliferation. Moreover, in the synovial fluid of patients with RA, the levels of AEA and 2-AG were found to increase and that of PEA was found to decrease, suggesting the prominent functional role of this pathway [32,66].

With reference to inflammatory lysophospholipids, the hydrolysis of circulating LPC contributed to the accumulation of the local production of LPA, which in turn amplified inflammation in synergy with TNF-α [33,34].

Ceramide and the activity of acid sphingomyelinase, which catalyzes the hydrolysis of sphingomyelin to ceramide, have been implicated in inflammatory arthritis. The inhibition of the activity of acid sphingomyelinase by genetic or pharmacological tools, such as amitriptyline, reduced the disease manifestations and levels of pro-inflammatory cytokines in a murine model [83]. A characteristic feature of RA is the disease fluctuation over the day, demonstrated by the changing symptoms and circulating markers of the inflammatory process. This joint inflammation oscillation was surprisingly linked to ceramide synthesis [84]. Furthermore, the sphingosine kinase-1 activity (SPHK1, which catalyzes the phosphorylation of sphingosine into S1P) increased at the sites of acute inflammation in both the murine model and patients. An increase in the production of S1P was hypothesized to cause the persistence of T-helper cells in the inflammation sites, contributing to the abnormal immune responses [35,36]. The expression of receptor 3 of S1P was associated with the development of autoimmunity as well as the increased downstream signaling and the production of cytokine IL-6 [85]. In contrast, the down-regulation of receptor 1 of S1P contributed to the regression of RA [86]. Elsewhere, it was postulated that acting on the mTOR pathway, S1P signaling could have a dual role: under physiological conditions, it maintains continuous bone turnover but leads to the pathogenesis of bone deformities during inflammation [87].

### 3.2. Type 1 Diabetes

Type 1 diabetes is characterized by the autoimmune-mediated destruction of pancreatic β-cells. The plasma lipid profile of patients with type 1 diabetes and murine models was characterized by the increase in the long-chain polyunsaturated triglycerides and the decreases in the long-chain lysophospholipids and cholesterol esters [88]. The corroborated hypothesis was the increase in the remodeling of both circulating lipoprotein and pro-inflammatory status in type 1 diabetes [88,89,90,91,92]. Notably, the levels of AA and AA-derived eicosanoids—such as thromboxane A2 (TXA2), leukotriene B4 (LTB4), PGD2, PGE2, 11-HETE, 12-HETE, 15-HETE, and 12-oxo-ETE—also decreased in a non-obese diabetic mouse, highlighting a reduced state of systemic inflammation [37].

The endocannabinoid system was shown to play a relevant role in the maintenance of effective immune responses in the gut, which is perpetually exposed to pathogenic insults [93]. Mononuclear phagocytes contributed to maintaining the equilibrium between inflammation and tolerance, which is a consequential state of hyporesponsiveness. In addition, AEA might protect against the development of autoimmune diabetes by modulating the immune-suppressive functions and the number of monocytes and macrophages [93].

When focusing on sphingolipidomics, an elevation in the levels of S1P was revealed in a cohort of patients with type 1 diabetes and in vivo models [7,8]. Other studies indicated that the pathogenesis of type 1 diabetes might be related to S1P pathways because it was involved in the disorders of T-cells migration and activation [94,95]. Moreover, in insulin-secreting cells, the low expression of endogenous S1P lyase contributed to the vulnerability of the cells to the toxicity brought by pro-inflammatory cytokines [96]. The role of S1P in inflammation and pathogenesis is not easy to understand. Autoimmune diabetes and especially its complications were associated with a dysmetabolism in the sphingolipids [7,9]. In particular, decreased plasma levels of very long-chain ceramide were associated with a significantly lower frequency of developing diabetes-related nephropathy and macroalbuminuria. Moreover, the reduction of sphingolipids containing fatty acid C24:1 was demonstrated and associated with a reduction in cardio- and neuro-protection [7,9].

### 3.3. Systemic Lupus Erythematosus

Systemic lupus erythematosus (SLE) is a disease of unknown etiology. SLE is more frequent in women than in men, and its symptomatology includes non-erosive and non-deforming arthritis, cutaneous rash, vasculitis, and other systemic manifestations [97,98]. Changes in the lipid composition in patients with SLE might be attributable to oxidation damages, mTORC1-dependent mitochondrial dysfunction [99], and cell death. In the pathophysiology of SLE, cell death concurred with the accrual in the production of autoantigens and autoantibodies [100]. Some alterations such as variations in the levels of plasmalogens and fatty acids could be associated with the changes in the lipid composition of the membrane of lymphocytes and with the deregulation of the immune system, including the abnormal recognition of autoantigens and enhanced production of antibodies. The uncontrolled production of reactive oxygen species (ROS) by neutrophils appeared at the onset and in the progression of SLE, thus causing increased oxidation in membrane lipids and the formation of other products such as isoprostanes [101,102]. To exemplify, the reaction of ROS with membrane lipids could lead to the formation of toxic lipidic hydroperoxides and their degradation products, which can further react with proteins and modify their structure and function [102]. The overproduction of ROS and lipid peroxidation products were related to the inflammatory status and therefore considered as a factor that, indeed, characterizes the disease [103].

Another piece of evidence is the high levels of glycosphingolipids in T lymphocyte membranes [104]. The disease did not modify the total content of circulating sphingolipids. However, it demonstrated an altered length of the FAs incorporated in SM and ceramide, with an increase of long-chain FAs and a decrease of very long-chain FAs [15]. Moreover, the plasma and serum levels of ceramide C24:1 increased owing to SLE renal complications. Therefore, ceramide C24:1 could be considered as a potential biomarker of lupus nephritis [10]. In particular, the levels of circulating ceramides and hexosylceramides were increased and sphingoid bases were decreased in SLE. These levels were associated with disease activity, and accordingly, they were normalized after immunosuppressive treatment [11]. Although the alteration in S1P expression was observed in different studies, its effects need to be further elucidated [10,11,105,106,107]. Sphingolipids profile modification could also occur in the vascular complication related to SLE, namely atherosclerosis [12].

Endocannabinoid modulation was demonstrated by higher 2-AG levels in patients with SLE compared with healthy subjects. The 2-AG increment was associated with disease regression and supported the hypothesis of its protective action against the pathogenesis of SLE [13]. However, another cohort of patients did not exhibit this biochemical alteration; therefore, 2-AG levels could be associated with the disease manifestation [108].

To conclude, SLE patients showed a notably altered lipid metabolism that is further demonstrated by a specific increase in the plasma concentrations of some LPEs, including 20:4 and 22:6 FA [14], and a decrease of LPC 18:2 [15].

### 3.4. Inflammatory Bowel Diseases

The term IBD is comprehensive of disabling, chronic inflammatory processes directed against intestinal mucosae, such as ulcerative colitis and Crohn’s disease (CD). IBDs are characterized by mucosa cell necrosis or the ulceration and infiltration of neutrophils into lesions. The disruption of epithelial barrier function observed in patients with IBD has been traditionally attributed to cytokines, but some studies have shed light on the role of PGE2 in paracellular regulation and mucosa impairment [16,109]. These showed that PGE2 binds receptors EP1–EP4 and then acts, via a Ca^2+^-mediated pathway, on myosin light chain kinase. The activity of the kinase included changing the transmembrane distribution of the tight junction proteins and the peri-junctional actin rings [16]. An increase in PGE2 mucosa levels was also demonstrated in Rodríguez-Lagunas et al.’s studies [16]. Moreover, the epithelial cells displayed the deregulation of the balance of the local levels of eicosanoids and endocannabinoids. An increase in the levels of HxA3, a pro-inflammatory and neutrophil chemoattractant eicosanoid, as well as a concomitant decrease in the anti-inflammatory endocannabinoids, were shown [18]. A common complication of CD is fibrosis, which includes the excessive deposition of extracellular matrix and the obstruction of the gut lumen. This complication was related to a differential DNA methylation that causes a decrease in the expression of mRNA encoding for prostacyclin synthase and an increase in that for prostaglandin D2 [19]. Plasma levels in IBD in the murine model showed impairment in the profile of eicosanoids, which promote inflammation and may induce carcinogenesis. In particular, in a murine model (IL-10(−/−)), EETs and DHETs underwent a decrease compared with the wild type [20].

In the gastrointestinal tract, endocannabinoids have been proposed to control the muscular propulsion, but they can also control several pathological functions. Studies have shown [21,110,111] that in animal models as well as in patients with ulcerative colitis, colon inflammation is accompanied by an increment of anandamide but not 2-AG. These findings were registered in mouse colon and biopsy samples from patients, confirming a possible role of the protective action of anandamide and its possible therapeutic employment [21]. Another study revealed a change in the gene expression in the macrophages and lymphocytes of non-classical endocannabinoid receptors including GPR55 and MGL. These components played an essential role in the regulation of the immune response toward intestinal and systemic inflammation [110]. After inflammatory stimuli, cannabinoid receptors could be modulated in their intestinal localization. In particular, CB2 increased in colonic epithelial cells, and CB1 increased in enteric neurons and the endothelium [111].

### 3.5. Multiple Sclerosis

MS is an autoimmune disease of the central nervous system that causes progressive neurologic disability. It is characterized by chronic inflammation that damages myelin axons and the myelin sheath [25].

A targeted lipidomic study performed on the spinal cords of experimental autoimmune encephalomyelitis (EAE) highlighted a metabolic switch, which determined an increase of the PGE_2_ pathway and a decrease of the PGD_2_, PGI_2_, and 5-LO pathways. Eicosanoid levels in the spleen and plasma were also measured; however, significant fluctuations were not found. These changes, coupled with an elevation in the expression of PGE2 receptors (EP1, EP2, and EP4), were correlated with clinical symptoms [112,113]. Moreover, a decrease in the serum concentration of neuroprotective lysophospholipids in patients with MS was shown. This decrease was attributed both to an impaired spleen homing of T-cells and to the demoting remyelination. However, LPA might also trigger pro-inflammatory cellular responses depending on the binding receptor and the source [22]. Nevertheless, whether LPA can be used as a biomarker of MS remains unclear. Two studies investigated LPA levels in a cohort of patients (*n* = 20); although an elevation of LPA serum levels in the patients compared with healthy controls was demonstrated, the possible role of LPA in the disease progression was not elucidated [23,24].

The membrane lipid abnormalities in lymphocytes and monocytes were attributable to impaired membrane fluidity along with the disease progression [114]. The results from this study showed that the membrane lipids of patients with MS and control subjects have no significant differences. However, a correlation was demonstrated between membrane fluidity—measured by lipid composition (phospholipids, fatty acids, and cholesterol)—and disease progression, estimated by the functional system score [114].

During MS, S1P levels were elevated in the central nervous system cell lineages. The S1P level in the EAE mice model affected with autoimmune EAE spinal cord was approximately twice that in the wild type, consistent with astrogliosis. Notably, the S1P levels decreased in S1P1 conditional null mutants [26].

### 3.6. Graves’ Disease

Graves’ disease is an autoimmune thyroid disease that represents one of the most common causes of hyperthyroidism. In addition to the signs and symptoms of hyperthyroidism, Graves’ disease is accompanied by a typical orbitopathy called Graves’ orbitopathy (GO). In particular, the orbital tissue of patients with GO showed elevated levels of S1P compared with the control samples. S1P was hypothesized to act as a chemoattractant for T-cells during disease progression [27]. The initial binding of T-cells activated the orbital fibroblasts via CD40, which caused an augmentation of S1P levels [27]. In addition, the role of S1P on adipogenesis and fibrosis [29,30] and in pro-inflammatory responses has been demonstrated. More specifically, IL-1β enhanced the expression of S1P receptors and sphingosine kinase in GO orbital fibroblasts; this in turn increased the expression of other pro-inflammatory mediators, including ICAM-1, COX-2, and IL-6 proteins [31].

## 4. Anti-Inflammatory Lipids and their Therapeutic Potential

Some recent works have focused on the functions of the precursors of eicosanoids: omega-3 polyunsaturated fatty acids (ω-3 PUFAs), EPA, and DHA. However, these precursors were proposed as potential candidates for the prevention or even treatment of some AIDs, such as type 1 diabetes, RA, SLE, and MS. Many of the beneficial effects of ω-3 PUFAs could be assigned to their anti-inflammatory properties coupled with the regulation of mTOR activity [115,116,117]. In particular, the anti-inflammatory properties of marine-derived ω-3 PUFAs could be related to a change in the fatty acid composition of the cell membranes [118] along with a decrease in the levels of eicosanoids, cytokines, and adhesion molecules. Another evidence of the anti-inflammatory properties of ω-3 PUFAs was the increasing levels of pro-resolving mediators [118,119]. In clinical trials, pro-resolving mediators both reduced inflammation—for example, decreased LTB4 [120]—and stabilized advanced atherosclerotic plaques in patients with RA [121]. Moreover, some symptomatic benefits could be obtained by combining paracetamol with fish oil (rich in PUFAs). This combination provided superior suppression of inflammatory PGE2 synthesis [122]. Nevertheless, note that information on ω-3 PUFAs supplementation based on clinical trials might be difficult to interpret owing to the differences in dose, duration, and drug interactions [119]. In a murine model of type 1 diabetes, dietary treatment with ω-3 PUFAs reduced the incidence of autoimmunity in pancreatic islets, modulated the differentiation of Th- and T-regulatory cells, and decreased the levels of pro-inflammatory mediators such as IFN-γ, IL-17, IL-6, and TNF-α [123]. In a murine model of colitis, EPA and DHA supplementation caused a significant increase in the levels of some anti-inflammatory eicosanoids. However, this change was not sufficient to alleviate colitis [124]. A study suggested that micronized PEA may be considered in relapsing–remitting MS to reduce the cutaneous adverse effects related to the subcutaneous administration of interferon (IFN)-β1 [125]. Lipoxins, resolvins, and protectins co-administered with aspirin might be useful in various rheumatological conditions [97]. To exemplify, the combination of ω-3 with non-steroidal anti-inflammatory drugs generated bioactive lipids that could be used downstream by leukocytes to counteract inflammation propagation [126]. Some results suggested that the administration of PPAR ligands such as 15d-PGJ2 may be a novel therapeutic strategy for MS because PPAR ligands efficiently reduced the severity of the inflammation by reducing both the secretion of encephalitogenic T-cells and cytokines and the consequent demyelination [127]. Moreover, EETs could be considered as a novel therapeutic agent for rheumatic disorders because they promoted tissue regeneration along with the attenuation of bone loss and osteoclast activity [128].

The stabilized cyclic phosphatic acid 2-carba-cPA (obtained by replacing one of the cyclic phosphate oxygen molecules with a methylene group at the sn-2 position) as well as its precursor, cyclic phosphatic acid, protected the oligodendrocytes by reducing mitochondrial apoptosis. This suggested that the modulation of LPA could be relevant for the treatment of demyelinating conditions [129]. Endocannabinoids can also display anti-inflammatory effects in different AIDs; however, their pharmacological potential is still debated. The exogenous supplementation of either COX-2 inhibitors [130], cannabinoid receptor agonists [65,66], or endocannabinoid degradation inhibitors [65,130,131,132] increased the levels of anti-rheumatic N-acylethanolamines. In the same way, in some diseases, the modulation of S1P pathway could be accomplished indirectly and not using exogenous S1P. S1P seemed to have a central role in interfering with an uncontrolled immune system by altering lymphocyte recruitment and the cellular adhesion processes. Moreover, blocking the S1P receptor function demonstrated efficacy in the treatment of MS. Fingolimod, the first non-selective S1PR agonist, and siponimod (BAF312), a selective S1PR1 agonist, have been approved to reduce inflammation in the treatment of relapsing–remitting and secondary progressive MS. Other S1PR modulators such as ponesimod (ACT128800), ozanimod (RPC1063), ceralifimod (ONO-4641), GSK2018682, and MT-1303 have been promising to reduce inflammatory demyelination and are also under clinical trials for MS treatment [133,134,135,136,137,138,139,140,141,142].

## 5. Concluding Remarks and Possible Roles of Biotechnology in the Prevention and Therapy of Autoimmune Inflammatory Diseases (AIDs)

Numerous new studies have proved the role of different functional lipids in the treatment of several AIDs, which are increasingly prevalent health problems among the global population. Nowadays, the prevalence of diagnosed autoimmune conditions is 7%, 6%, and 5% in the U.S., E.U. (average of a group of countries), and China, respectively [143]. Thus, further studies are urgently needed to shed light on the mechanisms and therapies of these diseases so as to counteract them.

It has been recognized that ω-3 PUFAs have anti-inflammatory properties and their presence in nutrition contributes to the prevention of many inflammatory diseases, independently from the mechanism of action. Nevertheless, for the development of drugs based on ω-3 PUFA derivatives for the cure of a given inflammatory disease, in-depth knowledge of the mechanism of action of the disease itself is required. Therefore, the administration or inclusion of ω-3 PUFAs in human diet appears as the most natural way to reduce AIDs insurgence. In particular, the availability of ω-3 PUFAs in human diet could dramatically change their benefits.

Although out of the scope of this review, emphasizing that biotechnology, in particular metabolic engineering, is increasingly adopted for the production of ω-3 PUFAs from different microbial strains is important. Analogously, plants have also been genetically engineered to produce high amounts of DHA and EPA. These achievements would undoubtedly allow a broader diffusion of these fatty acids, thus helping to improve the general health conditions. Human health will particularly benefit if these molecules are included as an essential part of diet [144,145,146].

An ideal tool to test the role of different types of functional lipids might be microfluidics tools combined with biotechnological techniques [147,148]. Such tools have allowed human cells and histological tissues to be cultivated and treated under strictly controlled conditions. These approaches, even if they are only at the initial stages of development, have already been proven as a strategic system to provide new insights into several areas of medicine, especially therapeutic devices with the role of organoids. From a pharmacological point of view, biotechnology could positively contribute to the production of the intermediates involved in the regulation of inflammatory diseases. This review provides an overview of AIDs and aims to encourage scientists to develop engineered microorganisms for the large-scale production of selected functional lipids or their derivatives.

## Figures and Tables

**Table 1:** Functional lipids and their corresponding role in autoimmune diseases.

Autoimmune Diseases	Lipids	Roles	Changes ^1^	References
Rheumatoid arthritis	11-HEPE, 12-HEPE and 15-HEPE (EPA-derived)	anti-inflammatory	up	[5]
	PGE2, 6,15-dk, dh, PGF1α, 12-HETE, 12LOX derived eicosanoids (AA-derived)	pro-inflammatory	up	[5]
	Resolving D1 and 17-HDoHE (DHA derived)	anti-inflammatory	down	[5]
	molecules from COX and LOX pathways	remodeling process of inflammation	up	[6,78]
	5-HETE and LTD4	remodeling process of inflammation	down	[6]
	PGE2 and 15-(S)-HETE	inflammatory stimulators	up	[17,79]
	PGD2	anti-inflammatory	up	[80]
	8-iso-PGF(2α) and 15-keto-dihydro-PGF(2α)	Oxidative stress and inflammation biomarkers	up	[81,82]
	Resolvin D3	resolution of inflammation	down	[28]
	AEA and 2-AG	neovascularization, cartilage, and bone demolition	up	[32]
	PEA	anti-inflammatory	down	[32]
	LPA	pro-inflammatory	up	[33,34]
	S1P	abnormal immune responses	up	[35,36]
Type 1 diabetes	thromboxane A2 (TXA2), leukotriene B4 (LTB4), PGD2, PGE2, 11-,12- and 15-HETE, and 12-oxo-ETE	impaired states of systemic inflammation	down	[37]
	S1P	immunomodulation	up	[7,8]
	omega-9 esterified sphingolipids	cardio- and neuro-protection	down	[7,9]
Systemic lupus erythematosus	Ceramide 24:1	biomarker of lupus nephritis	up	[10]
	Ceramides, hexosylceramides	associated with disease activity and vascular complication	up	[11,12]
	2-AG	disease regression	up	[13]
	Phospholipids (PE)	increased oxidative stress	down	[14,15]
Inflammatory bowel diseases	PGE_2_	paracellular regulation	up	[16]
	HxA3	pro-inflammatory, activates migration	up	[18]
	Endocannabinoid agonist on CB2	anti-inflammatory, inhibits migration	down	[18]
	prostaglandin D2 and prostacyclin	excessive deposition of extracellular matrix and obstruction of the gut lumen	down	[19]
	epoxyeicosatrienoic acids (EET) and dihydroxyeicosatrienoic acids (DHETs)	anti-inflammatory activity	down	[20]
	PGE2 and 5-HETE metabolites	pro-inflammatory	up	[20]
	Anandamide	Protective action	up	[21]
Multiple sclerosis	Lysophospholipids	demoting remyelination	down	[22]
	Lysophosphatidic acid (LPA)	Pro- and anti-inflammatory	up	[23,24]
	PGE_2_	Pro-inflammatory	up	[25]
	LTC4, LTB4, LTD4	anti-inflammatory	down	[25]
	S1P	astrogliosis	up	[26]
Grave’s disease	S1P	chemoattractant for T cells, pro-inflammatory, fibrosis and adipogenesis	up	[27,29,30,31]

^1^ This column reports if the lipids concentrations are incremented (up) or decremented (down) as a consequence of the biological response to the effects of the disease.

## Abbreviations

AIDs	autoimmune inflammatory diseases
COX	cyclooxygenases
LOX	lipoxygenases
AA	arachidonic acid
EPA	eicosapentaenoic acid
DHA	docosahexaenoic acid
GL	glycerophospholipids
LTs	leukotrienes
PGs	prostaglandins
TXs	thromboxanes
Rv	resolvins
AEA	anandamide
2-AG	2-arachidonoilglycerol
PEA	palmitoylethanolamide
OEA	O-arachidonoylethanolamine
LPA	lysophoshatidic acid
PA	phosphatidic acid
DAG	diacyglicerols
TAG	triacylglicerols
CDP-DAG	cytidine ciphosphate diacylglycerol
CL	cardiolipins
PI	phosphatidyl inositoles
LPI	lysophosphatidyl inositoles
PG	phosphatidyl glicerols
LPG	lysophosphatidyl glicerols
PE	phosphatidyl ethanolamines
LPE	lysophosphatidyl ethanolamines
PC	phosphatidyl cholines
LPC	lysophosphatidyl cholines
PS	phosphatidyl serines
LPS	lysophosphatidyl serines
GlcCer	glucosylceramide
LacCer	lactosylceramide
S1P	sphingosine 1-phosphate
GalCer	galactosylceramide
SM	sphingomyelins
Cer-1P	ceramide 1-phsosphate
EETs	Epoxyeicosatrienoic acids
DHETs	dihydroxyeicosatrienoic acids
HETEs	hydroxyeicosatetraenoic acids
CB	cannabinoid receptor
RA	rheumatoid arthritis
SLE	systemic lupus erythematosus
ROS	reactive oxygen species
IBD	inflammatory bowel disease
CD MS	Crohn’s disease multiple sclerosis
GO	called Graves’ orbitopathy
ω-3 PUFAs	omega-3 polyunsaturated fatty acids
